# Does gentle assisted pushing or giving birth in the upright position reduce the duration of the second stage of labour? A three-arm, open-label, randomised controlled trial in South Africa

**DOI:** 10.1136/bmjgh-2018-000906

**Published:** 2018-06-29

**Authors:** G Justus Hofmeyr, Joshua P Vogel, Mandisa Singata, Ndema Abu Habib, Sihem Landoulsi, A Metin Gülmezoglu

**Affiliations:** 1 Effective Care Research Unit (ECRU), Department of Obstetrics and Gynaecology at East London Hospital Complex (ELHC), University of the Witwatersrand, University of Fort Hare, Walter Sisulu University and Eastern Cape Department of Health, East London, South Africa; 2 UNDP/UNFPA/UNICEF/WHO/World Bank Special Programme of Research, Development and Research Training in Human Reproduction (HRP), Department of Reproductive Health and Research, World Health Organization, Geneva, Switzerland

**Keywords:** obstetrics

## Abstract

**Introduction:**

Gentle assisted pushing (GAP) is an innovative method of applying gentle, steady pressure to a woman’s uterine fundus during second stage of labour. This randomised trial evaluated GAP in an upright position, compared with upright position alone or routine practice (recumbent posture).

**Methods:**

An open-label, hospital-based, randomised trial was conducted in Eastern Cape, South Africa. Randomisation occurred following at least 15 min in second stage of labour. Participants were randomly assigned (1:1:1) using computer-generated block randomisation of variable size using opaque, sealed, numbered envelopes. Primary analysis was intention to treat. Participants were healthy, nulliparous, consenting women with a singleton pregnancy in cephalic presentation where vaginal birth was anticipated. The primary outcome was mean time from randomisation to birth.

**Results:**

1158 participants were randomly allocated to GAP (n=388), upright position (n=386) and routine practice (n=384), with no loss to follow-up. Baseline characteristics were largely similar. In the experimental arm, GAP was applied a median of two times (IQR 1.0–3.0). Women in upright position alone spent a median of 6 min (IQR 3.0–10.0) upright. Mean duration from randomisation to birth was not different across groups (mean (SD) duration: 24.1 (34.9) min in GAP group, 24.6 (30.5) min in upright group, 25.0 (39.3) min in routine practice group). There were no differences in secondary outcomes, except that at two sites maternal discomfort was greater for both GAP and upright position compared with routine practice; at the other sites there were no differences.

**Conclusion:**

No benefit was identified from GAP in the second stage; some women found the position uncomfortable. The use of fundal pressure should be limited to further research to determine techniques or settings in which it can safely assist vaginal birth. Women should be encouraged to assume the position they find most comfortable.

**Trial registration number:**

PACTR201502001034448.

**Key questions**

**What is already known?**

- Nine trials have been conducted previously on fundal pressure during the second stage of labour compared with no treatment—five trials (3057 women) of manual fundal pressure and four trials (891 women) of fundal pressure by means of an inflatable belt versus no fundal pressure. Most trials had design limitations, and none were able to blind women or staff to allocation.
- There is insufficient evidence to draw conclusions on the beneficial or harmful effects of fundal pressure (either manually or by inflatable belt) for mother or baby.
- Because of current widespread use of fundal pressure in clinical settings, and the potential for use in settings where other methods of assisted birth are not available, further good quality trials are needed to guide practice.

**What are the new findings?**

- This trial showed no clear benefit from a new technique of a gentle, controlled form of manual fundal pressure, nor of upright position alone in the second stage of labour. Some women found the upright position (with or without fundal pressure) uncomfortable, although findings were mixed.

**What do the new findings imply?**

- There is insufficient evidence of effectiveness or safety to support the use of fundal pressure, except in the context of research designed to determine whether specific techniques of fundal pressure in certain clinical situations may be beneficial and safe. Given the lack of clear benefits of specific postures in the second stage of labour, women may be encouraged to use the posture they find most comfortable.

## Background

Applying manual fundal pressure to expedite birth was originally described by Samuel Kristeller in the 1870s and was known as the Kristeller manoeuvre. While techniques vary, fundal pressure has been described as the application of pressure by the birth attendant to the uppermost part of a woman’s uterus, directed towards the birth canal, in an attempt to assist spontaneous vaginal birth.^1^ Under controlled conditions, this technique has been shown to increase intrauterine pressure.^2^ The Cochrane review on fundal pressure during the second stage of labour identified five trials (3057 women) of manual fundal pressure and four trials (891 women) of fundal pressure applied by an inflatable belt.^1^ The review concluded that there is currently insufficient evidence to draw conclusions on the beneficial or harmful effects of fundal pressure and further good quality trials are needed.

Despite the lack of evidence fundal pressure is used in many countries.^3–6^ It has been both recommended and condemned as a strategy for assisting birth, particularly for shoulder dystocia management.^7 8^ It is often applied routinely, or when there is evidence of prolonged second stage of labour, a need to expedite birth (eg, fetal distress), or for maternal medical conditions where prolonged pushing is contraindicated.^1^ It is also used to assist birth at Caesarean section. It is difficult to quantify or control the amount of force used—it can vary from gentle pressure with one hand, to vigorous force applied using the attendant’s whole weight. Use of rapid thrusting movements may cause sharp rises in intrauterine pressure and increase the risk of maternal and neonatal injury.

Reliable evidence on the benefits and/or harms of fundal pressure applied in a safe and controlled manner can have important implications for clinical practice. We therefore developed and evaluated the gentle assisted pushing (GAP) technique, a gentle fundal pressure manoeuvre performed by a skilled, trained birth attendant (midwife or doctor) with the woman in the upright position. In 2013, we conducted a two-arm pilot randomised controlled trial (GAP vs routine practice) of 120 healthy nulliparous women in South Africa with singleton, cephalic pregnancies using this technique. The pilot study demonstrated GAP is feasible in our clinical setting and further investigation of safety and efficacy was warranted.^9^

In assessing GAP’s effect on duration of labour and other outcomes, it is important to account for the effect of upright position in the second stage of labour, which may facilitate vaginal birth by enabling the mother to bear down more efficiently, as well as tilting the pelvis to a more favourable orientation. A Cochrane review has shown that in women without epidural anaesthesia, the upright position might reduce duration of second stage (though not in the trials with lower risk of bias) and reduce rates of assisted birth, episiotomy and abnormal fetal heart rate patterns, while second-degree perineal tears and estimated blood loss over 500 mL may increase.^10^ A recent randomised trial in women with low-dose epidural analgesia found that spontaneous vaginal birth was less frequent with upright posture.^11^ The objective of this trial was to compare GAP in an upright position to upright position alone, or routine practice (supine/recumbent position) on duration of second stage of labour and neonatal and maternal outcomes.

## Methods

The study protocol was registered with the Pan African Clinical Trials Registry (PACTR201502001034448), and published previously (online supplementary appendix 1).^12^ The trial was conducted in accordance with Good Clinical Practice (GCP) standards and monitored by a data and safety monitoring board (DSMB), and is reported according to Consolidated Standards of Reporting Trials guidance (online supplementary appendix 2).^13^

10.1136/bmjgh-2018-000906.supp1 Supplementary data

10.1136/bmjgh-2018-000906.supp2 Supplementary data

### Study design and participants

The GAP study was a multicentre, randomised, open-label clinical trial with three parallel arms. The study was conducted at four sites across three hospitals in the Eastern Cape, South Africa. Frere Maternity Hospital (midwife-led obstetric unit and obstetrician-led care unit at Frere were separate study sites) and Cecilia Makiwane Hospital are tertiary hospitals in East London, South Africa (with 6840 and 4800 births annually, respectively). Butterworth Hospital is a nearby district hospital with 4800 births annually.

Women were informed about the trial in the antenatal period or latent phase. Eligible women were nulliparous, at least 18 years of age, at 35 or more weeks of gestation with a singleton, cephalic pregnancy, in whom a vaginal birth was anticipated. Women with chronic medical conditions (including heart disease, epilepsy, hypertension, diabetes mellitus and renal disease) and obstetric complications (including hypertensive disorders of pregnancy, cephalopelvic disproportion, antepartum haemorrhage, intrauterine growth restriction and fetal distress) were not eligible. If eligible, the informed consent process was initiated.

### Randomisation and allocation

Consenting women who were undelivered following at least 15 min of expulsive efforts in the second stage of labour were randomised by research staff by opening the next sequentially numbered, sealed, opaque envelope containing the participant’s group assignment. The randomisation sequence and envelopes were prepared by the WHO data manager, using a computer-generated random sequence in balanced blocks of variable size, in a ratio of 1:1:1. It was not possible to blind participants and staff to allocation after randomisation.

Participants were randomly allocated to one of the three intervention groups: GAP (in upright position); upright position only; and routine practice (recumbent/supine position). Other than the allocated intervention, routine clinical practice was otherwise used in all arms, including fetal heart monitoring and routine postpartum oxytocin use. Women were encouraged to adopt the allocated position, however at any stage of labour, change in position, oxytocin administration, instrumental birth or caesarean section could be used, as per decisions of the responsible clinician. It was anticipated that some women might find their allocated position uncomfortable. Participants were specifically assured of their right to decline or to withdraw from the study for any reason, and that it would not affect the medical care to which she was entitled. There was no payment for participation.

#### Arm 1—GAP

The GAP method was specifically developed to avoid vigorous fundal pressure. The woman is assisted to assume an upright kneeling or squatting position on the bed. The trained birth attendant (midwife or doctor) kneels on the bed or stands behind the woman, wraps her arms around her (passing below her axillae) and places both open palms, overlapping, on the uterine fundus. Steady (firm yet gentle) pressure is applied in the long axis of the uterus during contractions for a maximum of 30 s, with at least 30 s rest before the next application. The relative positions of woman and attendant ensure that excessive force cannot be used. Labour ward and study staff at all sites underwent initial and refresher training in applying GAP, using standardised training with video demonstrations and simulation. An example of the GAP technique is available in online supplementary appendix 3.

10.1136/bmjgh-2018-000906.supp3 Supplementary data

#### Arm 2—Upright position

Participating women were encouraged to assume an upright position (kneeling, squatting or crouching) during the second stage of labour, with no fundal pressure. When the baby’s head crowned, the birth attendant could choose to move the woman to a recumbent position for the birth, if required.

#### Arm 3—Routine practice

The current position as practised in the participating sites was used (recumbent/supine). If women remained undelivered 30 min after randomisation, fundal pressure or changes in position could be used according to routine local practice. Additional procedures or treatments at the discretion of the attendant were recorded in the participants’ records.

Electronic fetal heart rate monitoring was not routinely used in uncomplicated second-stage pregnancies in the participating hospitals. The fetal monitoring protocol was for auscultation of the fetal heart rate after each contraction. If electronic monitoring was used, the GAP procedure does not interfere as the monitor is positioned on the anterior aspect of the uterus while pressure is applied to the fundal aspect.

### Study outcomes

The primary outcome was defined as mean time (minutes) from randomisation to birth. Secondary outcomes were: no spontaneous birth within 15 min of randomisation, operative birth (ie, birth by vacuum, forceps or caesarean section) and the use of episiotomy or perineal trauma (defined as second or higher degree perineal tear). Neonatal outcomes included: cord blood pH <7.2 or lactate ≥8 mmol/L (measured using neonatal intensive care unit blood gas analysers, and/or handheld strip lactate tests), Apgar score less than 7 at 5 min, neonatal injury, neonatal encephalopathy, admission to neonatal high care nursery for >24 hours and neonatal death. Maternal outcomes were discomfort and any adverse events. Maternal discomfort was assessed by asking women how comfortable they were during labour after randomisation (comfortable, uncomfortable or very uncomfortable).

Reporting of adverse events and serious adverse events was done using standard forms and reported to the DSMB on an expedited basis. Women and neonates were followed up until discharge. Any adverse events were followed up until resolution.

### Data management, sample size and statistical analysis

All data were entered into paper study forms. Completed forms were checked by another staff member against the original patient records for correctness. The data were double-entered into OpenClinica, a web-based, GCP-compliant data management system. Validation and consistency checks ensured accuracy and completeness of entered data.

The sample size was powered to detect a reduction in the primary outcome of mean time (minutes) from randomisation to birth of ≥3 min, based on Api *et al*^14^ who reported a time from randomisation to birth of 23.1 min (SD 12.2 min) in the control group (n=56), and 18.6 min (SD 9.5 min) in the fundal pressure group (n=34). We assumed that upright position alone would likely lie between these two (ie, approximately 20.0 min). We assumed a power of 90% and alpha of 95%. Using the Bonferroni rule to control for multiplicity, alpha is divided by the number of comparisons (0.05/3≈0.02). The study therefore required 347 women in each arm, that is, 1041 women for all three arms. To allow for 10% non-compliance, we aimed to recruit 382 in each arm, for a total sample size of 1146 women.

The statistical analysis plan was finalised before data analysis. An interim analysis was conducted after approximately 50% of participants were randomised; results reviewed by the DSMB who recommended that the trial continue. The primary analysis (intention to treat, ITT) was based on all participants with outcome data available as per their original allocation, regardless of compliance. Baseline characteristics for the randomised population were tabulated. Frequencies and percentages were reported for categorical outcome variables, while for continuous outcome variables the number of participants, means and SD were reported. Where the distribution was non-normal, medians, IQR, minima and maxima were reported.

In the event the primary outcome distribution was approximately normal, we planned to use crude mean (SD), and analysis of variance (ANOVA) regression models to compare the effect size between the three treatment groups, adjusted for centre. However, the data were non-normal (right skewed), and the natural logarithm (log) of the primary outcome was approximately normally distributed, hence we used a log transformation. In case of a statistically significant difference between the three treatment groups by the ANOVA test, additional pairwise tests were planned. The non-parametric Kruskal-Wallis test was used to compare differences in crude median time to birth, while Kaplan-Meier (K-M) right-censored survival analysis was used to analyse and compare median time to birth, as well as the rates between treatment groups, with significance tested using the log-rank p value stratified for centre.

For the secondary outcomes, log-binomial regression modelling was used to compare the risk of the outcome between treatment groups, adjusted for centre. Where the log-binomial model did not converge, a modified Poisson regression modelling with a robust variance was used.^15^ The crude proportion of severe adverse events (SAE) per randomised women was computed. A Poisson distributed generalised estimating equation model, with an independent working correlation structure, was used to estimate adjusted incidence rates of SAEs (expressed as events per 100 woman-days of observations, postrandomisation) as well as adjusted incidence rate ratios. The simpler independent correlation structure was chosen as some participants had more than one SAE, and also due to independency between reported SAEs per participant. Two-sided tests and 5% significance levels were used and 95% CIs for all relevant parameters. Statistical analysis was conducted using SAS/STAT software, V.9.4 of the SAS System for Windows.^16^ The graphs were plotted using R software.^17^

## Results

**Figure 1:**
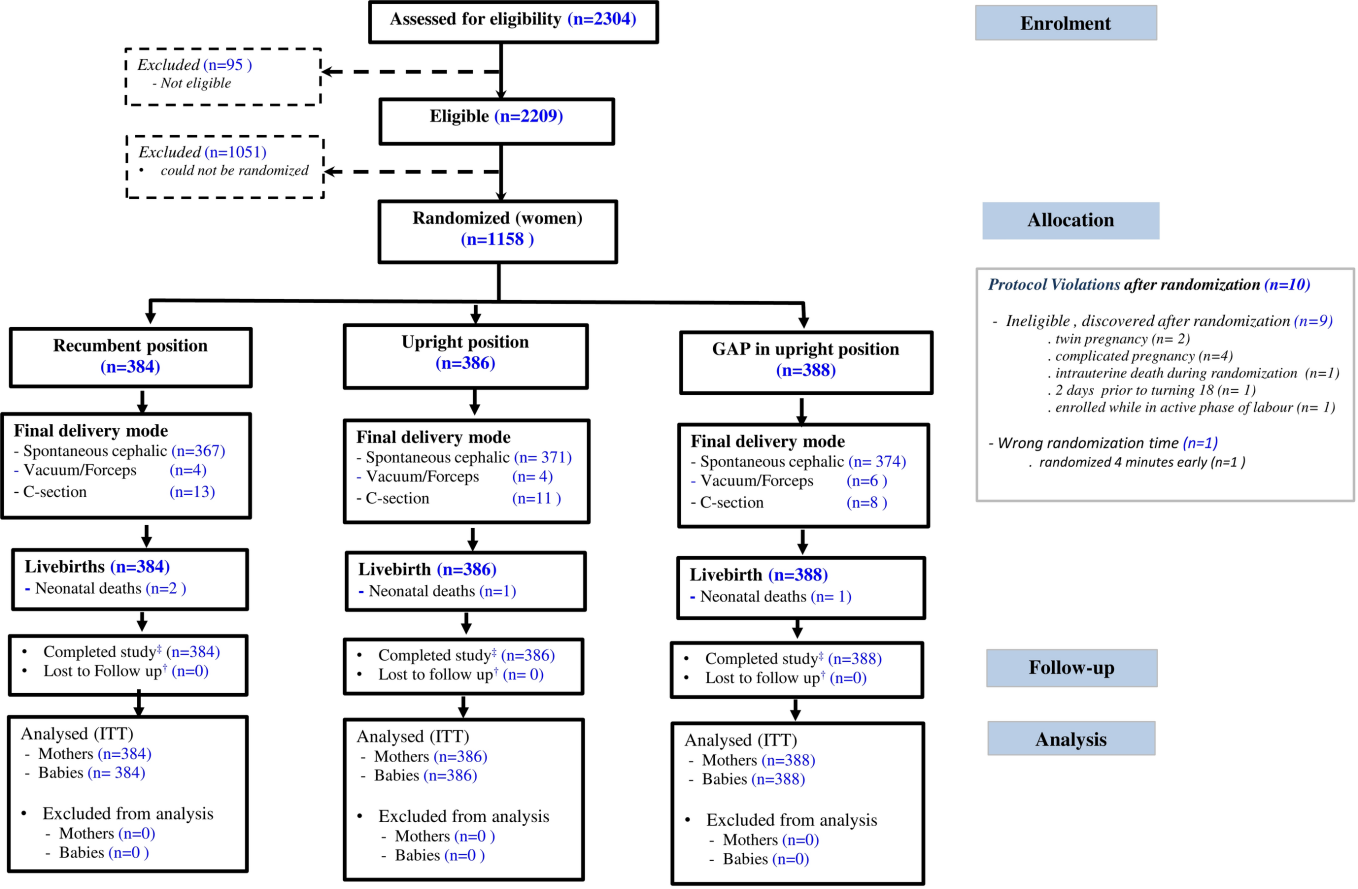
Consolidated Standards of Reporting Trials (CONSORT) flow diagram. ‡Completed study, if followed up from randomisation to maternal discharge from maternity unit (with non-missing date of discharge from maternity clinic). †Lost to follow-up is considered if occurred in the period following randomisation and prior to mom being discharged from maternity clinic. GAP, gentle assisted pushing; ITT, intention to treat.

Between 12 March 2015 and 11 August 2017 we randomly allocated 1158 women to GAP in upright position (n=388), upright position (n=386) and routine practice (n=384) (see figure 1). Ten protocol violations occurred after randomisation. No women or babies were lost to follow-up, hence the ITT analysis population included 1158 women and 1158 babies. The trial ended as the predetermined sample size target had been reached.

Baseline characteristics were largely similar across all three arms—overall, the median gestational age was 38 weeks and 21.8% of all women were HIV positive (table 1). At baseline, most women were in spontaneous labour (5.3% had labour induced) and 64.4% had not received pain relief medication. Labour augmentation in the second stage (prior to randomisation) was different between groups (5.9% in GAP, 3.1% in upright position, 2.6% in routine group). In the GAP group, GAP was applied a median of two times (IQR 1.0–3.0), and at least once in 320 women (82.5%), compared with four women (1.0%) in the upright group and five women (1.3%) in the routine practice group (table 2). Women in the GAP group spent a median of 5.0 min (IQR 3.0–9.0) in the upright position compared with 6.0 min (IQR 3.0–10.0) for the upright position group and 0.0 min (IQR 0.0–0.0) for the routine practice group. Overall, over 95.6% of women gave birth by spontaneous, cephalic vaginal birth (table 3).

**Table 1:** Baseline data

	GAP in upright position n=388			Upright position n=386			Routine practice n=384		
	n	Mean	SD	n	Mean	SD	n	Mean	SD
Age (years)	388	21.9	3.6	386	21.8	3.4	384	21.7	3.2
Gestational age (completed weeks)	388	37.8	1.6	386	37.8	1.6	384	37.9	1.7
Weight (kg)	386	72.8	13.7	385	73.5	13.6	381	73.1	13.9
Height (cm)	382	159.3	6.9	381	159.8	7.7	376	158.8	7.9
BMI (kg/m^2^)	381	28.7	5.2	381	28.8	5.4	374	28.9	5.2

	N	n	%	N	n	%	N	n	**%**
Obese (BMI ≥30.0 kg/m^2^)	381	143	37.5	381	140	36.8	374	140	37.4
Taking any medication	388	92	23.7	386	76	19.7	384	76	19.8
HIV positive	388	95	24.5	386	79	20.5	384	79	20.6
Labour was induced	388	22	5.7	386	18	4.7	384	21	5.5
Labour augmentation*									
Labour augmentation in first stage	387	25	6.5	386	24	6.2	384	23	6.0
Labour augmentation in second stage	387	23	5.9	385	12	3.1	384	10	2.6
Analgesia*									
None	386	249	64.5	386	257	66.6	383	240	62.7
Epidural	386	3	0.8	386	2	0.5	383	0	0.0
Spinal†	386	0	0.0	386	0	0.0	383	0	0.0
Epidural/spinal†	386	0	0.0	386	0	0.0	383	0	0.0
Injectable analgesic	386	134	34.7	386	127	32.9	383	143	37.3

*Prior to randomisation.

†All 15 cases where spinal or epidural/spinal was used were women who delivered by caesarean section (CS).

BMI, body mass index; GAP, gentle assisted pushing.

**Table 2:** Treatment adherence

	GAP in upright position n=388			Upright position n=386			Routine practice n=384		
	n	Median	Min, max	n	Median	Min, max	n	Median (IQR)	Min, max
Time spent in upright position (min)	387	5.0 (3.0, 9.0)	0.0, 8.0	386	6.0 (3.0, 10.0)	0.0, 58.0	379	0.0 (0.0, 0.0)	0.0, 50.0
Total number of contractions	388	3.0 (2.0, 5.0)	1.0, 14.0	386	3.0 (2.0, 5.0)	1.0, 17.0	384	3.0 (2.0, 4.0)	1.0, 15.0
Number of times GAP applied	386	2.0 (1.0, 3.0)	0.0, 9.0	383	0.0 (0.0, 0.0)	0.0, 2.0	384	0.0 (0.0, 0.0)	0.0, 3.0

	N	n	%	N	n	%	N	n	**%**
GAP was applied at least once	386	320	82.5	383	4	1.0	384	5	1.3
GAP applied only during contractions	320	294	91.9	4	4	100.0	5	5	100.0
GAP applied for no more than 30 s at a time	320	294	91.9	4	4	100.0	5	5	100.0

GAP, gentle assisted pushing.

**Table 3:** Birth outcomes

	Routine practice n=384			Upright position n=386			GAP in upright position n=388		
	N	n	%	N	n	%	N	n	%
Final mode of birth									
Spontaneous cephalic	384	367	95.6	386	371	96.1	388	374	96.4
Vaginal breech	384	0	0.0	386	0	0.0	388	0	0.0
Vacuum or forceps	384	4	1.0	386	4	1.0	388	6	1.5
Caesarean section	384	13	3.4	386	11	2.9	388	8	2.1
Reasons for operative birth									
Poor progress	17	4	23.5	15	6	40.0	14	5	35.7
Fetal distress	17	1	5.9	15	1	6.7	14	1	7.1
Other	17	12	70.6	15	8	53.3	14	8	57.1

GAP, gentle assisted pushing.

Data on the primary outcome were available for all women. The median duration from randomisation to birth was similar (14 min) in all three arms (table 4). Significance testing (of crude, natural log and ANOVA model adjusted for study centre) showed no difference between the three arms. The adjusted difference in mean log duration from randomisation to birth showed that GAP did not have an effect compared with routine practice (0.04 log min, 95% CI −0.14 to 0.20), nor when compared with upright position (−0.11 log min, 95% CI −0.28 to 0.06). Compared with routine practice, upright position also did not meaningfully reduce time to birth (0.14 log min, 95% CI −0.03 to 0.31) (online supplementary appendix 4: table 1). The K-M survival analysis did not show substantive differences between arms (online supplementary appendix 4: figure 1 and table 2). We also analysed the primary outcome among women with spontaneous birth only (1112 women), and results were similar.

10.1136/bmjgh-2018-000906.supp4 Supplementary data

**Table 4:** Primary outcome: duration from randomisation to birth in minutes

		GAP in upright position	Upright position	Routine practice
Number of women		388	386	384
Crude estimate of duration from randomisation to birth (min)	Mean (SD)	24.1 (34.9)	24.6 (30.5)	25.0 (39.3)
	Median (IQR)	14 (6, 29)	14 (9, 29)	14 (8, 25)
	Min, max	(0, 435)	(1, 519)	(0, 519)
	Kruskal-Wallis test	0.33		
Crude estimate of natural log (duration from randomisation to birth)	Mean (SD)	2.58 (1.23)	2.69 (1.02)	2.55 (1.36)
	Median (IQR)	(−6.91, 6.08)	(0.0, 5.63)	(−6.91, 6.25)
	Min, max	(−6.91, 6.08)	(0.0, 5.63)	(−6.91, 6.25)
	Kruskal-Wallis test	0.33		
Adjusted estimate* using ANOVA model: natural log (duration from randomisation to birth)	Mean (SE) 95% CI	2.53 (0.07) 95% CI (2.40 to 2.66)	2.64 (0.07) 95% CI (2.50 to 2.77)	2.49 (0.07) 95% CI (2.36 to 2.63)
	F-test	0.23		

*Adjusted for centre.

ANOVA, analysis of variance; GAP, gentle assisted pushing.

**Figure 2:**
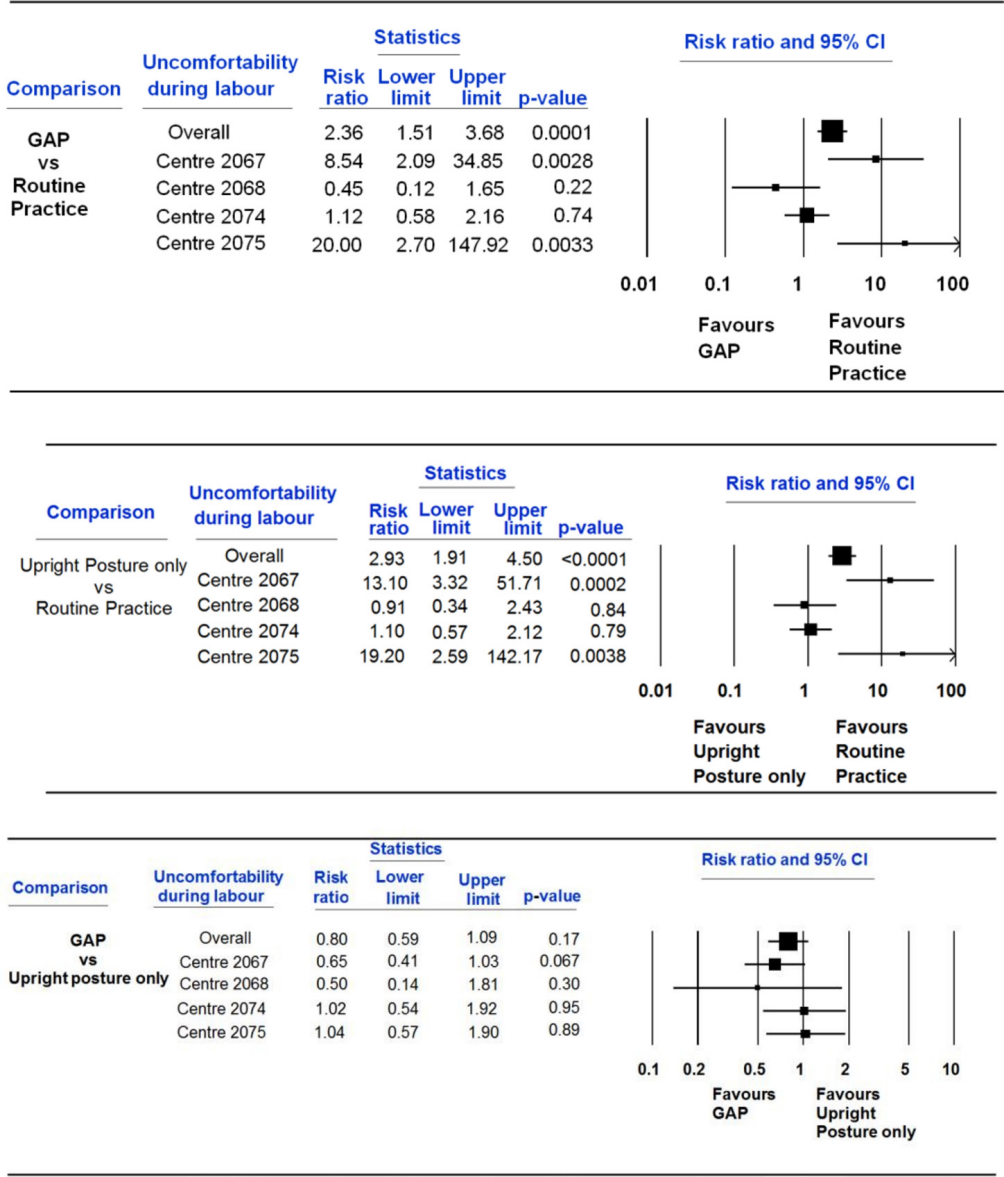
Mother was uncomfortable or very uncomfortable during labour. GAP, gentle assisted pushing.

Table 5 reports the effects on maternal and newborn secondary outcomes; several outcomes had few or zero events. There were four neonatal deaths in total—one each in the GAP and upright position arms, and two in the routine practice arm. No substantive differences were seen, except for differences in maternal discomfort during second stage of labour, which were inconsistent between sites (figure 2). At two sites, discomfort was greater for both GAP and upright position compared with routine practice and similar comparing GAP to upright position, while at the other two sites there were no differences between the groups. Given the differences in labour augmentation at baseline, we analysed primary and secondary outcomes adjusted for centre and augmentation in second stage of labour (prior to randomisation), however results were similar (data not shown).

**Table 5:** Secondary outcomes

	GAP in upright position n=388			Upright position n=386			Routine practice n=384			P values	Adjusted risk ratio* (95% CI)		
	N	n	%	N	n	%	N	n	%		GAP versus routine practice	Upright versus routine practice	GAP versus upright
Maternal outcomes													
No spontaneous birth within 15 min	388	154	39.7	386	169	43.8	384	165	43.0	0.47	0.91 (0.77 to 1.08)	1.01 (0.86 to 1.19)	0.90 (0.76 to 1.06)
Operative birth (vacuum to forceps or caesarean section)¶	388	14	3.6	386	15	3.9	384	17	4.4	0.84	0.91 (0.77 to 1.08)	1.01 (0.86 to 1.19)	0.90 (0.76 to 1.06)
Second-degree or higher perineal tear	385	27	7.0	384	25	6.5	383	21	5.5	0.67	1.28 (0.74 to 2.20)	1.19 (0.68 to 2.07)	1.08 (0.64 to 1.81)
Episiotomy	386	79	20.5	384	81	21.1	383	86	22.5	0.79	0.91 (0.69 to 1.19)	0.93 (0.71 to 1.22)	0.97 (0.74 to 1.28)
Episiotomy or second-degree or higher perineal tear	386	105	27.2	384	104	27.1	383	107	27.9	0.96	0.97 (0.77 to 1.22)	0.97 (0.77 to 1.22)	1.002 (0.79 to 1.26)
Mother was uncomfortable or very uncomfortable during second stage of labour	378	54	14.3	378	64	16.9	383	24	6.3	<0.0001	2.36 (1.51 to 3.67)	2.93 (1.91 to 4.51)	0.80 (0.59 to 1.09)
Labour augmented in the second stage to after randomisation	387	13	3.4	386	10	2.6	384	6	1.6	0.28	2.11 (0.82 to 5.44)	1.32 (0.59 to 2.93)	1,60 (0.59 to 4.33)
Additional management required for postpartum haemorrhage	388	9	2.3	386	8	2.1	384	7	1.8	0.89	1.25 (0.47 to 3.32)	1.12 (0.41 to 3.06)	1.12 (0.44 to 2.85)
Neonatal outcomes													
Cord blood pH <7.2	49	20	40.8	49	20	40.8	52	18	40.8	0.76	1.24 (0.75 to 2.05)	1.15 (0.70 to 1.90)	1.07 (0.67 to 1.74)
Cord blood pH <7.2 or high lactate (≥8 mmol/L)	55	29	52.7	60	32	53.3	59	26	44.1	0.57	1.06 (0.80 to 1.41)	1.06 (0.80 to 1.40)	1.00 (0.75 to 1.34)
5 min Apgar score <7	380	8	2.1	377	4	1.1	379	5	1.3	0.47	1.62 (0.53 to 4.88)	0.81 (0.22 to 3.00)	1.98 (0.60 to 6.51)
Neonatal trauma	388	0	0.0	386	0	0.0	384	2	0.5	0.11†	NA‡	NA‡	NA‡
Neonatal encephalopathy	388	3	0.8	386	1	0.3	384	5	1.3	0.21‡	0.59 (0.14 to 2.43)	0.20 (0.02 to 1.69)	2.96 (0.31 to 28.23)
Admission to NICU for >24 hours	388	3	0.8	386	5	1.3	384	8	2.1	0.25‡	0.38 (0.10 to 1.40)	0.62 (0.21 to 1.88)	0.60 (0.15 to 2.49)
Composite adverse neonatal outcome§	388	29	7.5	386	27	7.0	384	33	8.6	0.69	0.86 (0.54 to 1.38)	0.82 (0.51 to 1.32)	1.05 (0.64 to 1.72)
Neonatal death	388	1	0.3	386	1	0.3	384	2	0.5	0.70‡	NA‡	NA‡	NA‡

*Adjusted for centre.

†Fisher’s exact test.

‡Too few observations.

§This composite neonatal outcome includes: trauma, encephalopathy, cord blood pH <7.2, 5 min Apgar <7 or NICU admission >24 hours.

¶Operative birth includes caesarean section, vacuum or forceps.

GAP, gentle assisted pushing; NA, not applicable; NICU, neonatal intensive care unit.

In total, 33 maternal SAEs occurred—24 in the GAP arm, 3 in the upright position arm and 6 in the routine practice arm. While SAEs were significantly different overall, there were few cases per SAE category, and no obvious pattern of cause (online supplementary appendix 4: table 3). Only one SAE was considered possibly related to the intervention (second-degree perineal laceration in the GAP arm). A total of 57 neonatal SAEs occurred—23 in the GAP arm, 15 in the upright arm and 19 in the routine practice arm. There were three cases of neonatal encephalopathy in the GAP arm, one in the upright position arm and five in the routine care arm.

## Discussion

Given the extensive use of fundal pressure during childbirth globally and the vehement opinions both for and against its use, there is remarkably little robust evidence available on its benefits or risks.^1^ While four studies of fundal pressure applied by an inflatable abdominal belt have shown some reduction in duration of second stage of labour but increased anal sphincter injury, we found only five previous randomised trials of manual fundal pressure of variable quality, which showed no evidence of benefit. Our study also did not show any benefit with manual fundal pressure in the second stage. There are two possible reasons: that the method is indeed ineffective, or that the research process was insufficiently robust to demonstrate benefit. Possible inadequacies include that the women selected were not at sufficiently high risk of prolonged second stage for the intervention to change outcomes, or that the procedure was not applied effectively. Our findings do not exclude the possibility that a more robust form of fundal pressure may be effective.

The Cochrane review on the effect of upright posture alone on second stage of labour (without epidural analgesia) identified 30 trials of 9015 women, however the risk of bias was variable and the findings inconsistent.^10^ Overall, assisted deliveries, episiotomies and abnormal fetal heart rate patterns were reduced, while second-degree perineal tears and blood loss were increased and caesarean sections were similar between groups. The duration of second stage of labour was reduced overall, but no reduction was found in 12 of 21 studies. Among studies assessed as low risk of bias, the reduction was not statistically significant. Our findings are consistent with the lack of effect found in the trials with low risk of bias.

In our trial, there was a trend to fewer neonatal poor outcomes in both upright posture groups compared with the routine care group. While numbers were small and no statistical significance was reached, better neonatal outcomes would be consistent with the finding of fewer abnormal fetal heart rate patterns seen in the Cochrane review.^10^ Without clear evidence to support one maternal position over another, these results suggest that (in the absence of other clinical indications) women may adopt their preferred position for birth. While this study and other studies in the second stage of labour have been too small to assess the risk of rare adverse events, indirect evidence from large pregnancy posture studies suggests that the supine position may be harmful for babies.^18 19^

### Strengths and limitations

This pragmatic clinical trial had some limitations. The nature of the intervention means that allocation cannot be concealed, which can potentially bias results. While adherence was generally good, women were able to take an alternative birth position if they preferred. Notably, 17.5% of women in the GAP group did not have GAP applied, either due to birth occurring soon after randomisation, or the woman or attendant declining the intervention. While our results suggest that maternal discomfort may be greater in upright positions, this should be interpreted with caution given the variability between study sites. The reason for these differences is not known, however questions relating to discomfort may have been interpreted differently. The SAE data should also be considered with caution. As an unblinded trial, there is the risk of differential SAE reporting in different arms. In addition, while all events met the SAE technical criteria, most were relatively minor clinical events that resolved completely.

## Conclusion

We have not found evidence of benefit for a new technique of controlled, sustained fundal pressure in the second stage of labour. Our study was too small to address rarer safety outcomes. Use of fundal pressure in the second stage of labour should be limited to further research to determine whether there are techniques or clinical settings in which fundal pressure can be shown to safely assist vaginal birth. Our study did not show benefit of a supported upright position in second stage, and many women found the position uncomfortable. In the absence of clear evidence of benefits for specific positions, women should be encouraged to assume the position they find most comfortable.

## References

[1] Hofmeyr GJ, Vogel JP, Cuthbert A, et al Fundal pressure during the second stage of labour. Cochrane Database Syst Rev 2017;3:CD006067 10.1002/14651858.CD006067.pub3 28267223 PMC6464399

[2] Buhimschi CS, Buhimschi IA, Malinow AM, et al The effect of fundal pressure manoeuvre on intrauterine pressure in the second stage of labour. BJOG 2002;109:520–6. 10.1111/j.1471-0528.2002.01399.x 12066941

[3] Miller S, Cordero M, Coleman AL, et al Quality of care in institutionalized deliveries: the paradox of the Dominican Republic. Int J Gynaecol Obstet 2003;82:89–103. discussion 87-8 10.1016/S0020-7292(03)00148-6 12834953

[4] Goldman N, Glei DA Evaluation of midwifery care: results from a survey in rural Guatemala. Soc Sci Med 2003;56:685–700. 10.1016/S0277-9536(02)00065-5 12560004

[5] Declercq ER, Sakala C, Corry MP, et al Listening to Mothers II: Report of the Second National U.S. Survey of Women’s Childbearing Experiences: Conducted January-February 2006 for Childbirth Connection by Harris Interactive(R) in partnership with Lamaze International. J Perinat Educ 2007;16:15–17. 10.1624/105812407X244778 18769522 PMC2174391

[6] de Leeuw JW, Vierhout ME, Struijk PC, et al Anal sphincter damage after vaginal delivery: relationship of anal endosonography and manometry to anorectal complaints. Dis Colon Rectum 2002;45:1004–10. 12195182 10.1007/s10350-004-6351-5

[7] Penney DS, Perlis DW Shoulder dystocia: when to use suprapubic or fundal pressure. MCN Am J Matern Child Nurs 1992;17:34–6. 1738307 10.1097/00005721-199201000-00012

[8] Gross SJ, Shime J, Farine D Shoulder dystocia: predictors and outcome. A five-year review. Am J Obstet Gynecol 1987;156:334–6. 3826169 10.1016/0002-9378(87)90278-x

[9] Novikova N, Mshweshwe N, Xoliswa W, et al O688 A new method of controlled fundal pressure during the second stage of labour: Randomized pilot study. International Journal of Gynecology & Obstetrics 2009;107(Suppl 2):S290 10.1016/S0020-7292(09)61061-4

[10] Gupta JK, Sood A, Hofmeyr GJ, et al Position in the second stage of labour for women without epidural anaesthesia. Cochrane Database Syst Rev 2017;5:CD002006 10.1002/14651858.CD002006.pub4 28539008 PMC6484432

[11] Epidural and Position Trial Collaborative Group. Upright versus lying down position in second stage of labour in nulliparous women with low dose epidural: BUMPES randomised controlled trial. BMJ 2017;359:j4471. 29046273 10.1136/bmj.j4471 PMC5646262

[12] Hofmeyr GJ, Singata M, Lawrie T, et al A multicentre randomized controlled trial of gentle assisted pushing in the upright posture (GAP) or upright posture alone compared with routine practice to reduce prolonged second stage of labour (the Gentle Assisted Pushing study): study protocol. Reprod Health 2015;12:114 10.1186/s12978-015-0105-9 26669766 PMC4681100

[13] Schulz KF, Altman DG, Moher D, et al CONSORT 2010 statement: updated guidelines for reporting parallel group randomised trials. PLoS Med 2010;7:e1000251 10.1371/journal.pmed.1000251 20352064 PMC2844794

[14] Api O, Balcin ME, Ugurel V, et al The effect of uterine fundal pressure on the duration of the second stage of labor: a randomized controlled trial. Acta Obstet Gynecol Scand 2009;88:320–4. 10.1080/00016340902730326 19172441

[15] Zou G A modified poisson regression approach to prospective studies with binary data. Am J Epidemiol 2004;159:702–6. 10.1093/aje/kwh090 15033648

[16] Institute S. The SAS system for Windows, Release 9.4. Cary, North Carolina: SAS Institute, 2014.

[17] R Core Development Team. R: a language and environment for statistical computing. Vienna, Austria: R Foundation for Statistical Computing, 2017.

[18] McCowan LME, Thompson JMD, Cronin RS, et al Going to sleep in the supine position is a modifiable risk factor for late pregnancy stillbirth; Findings from the New Zealand multicentre stillbirth case-control study. PLoS One 2017;12:e0179396 10.1371/journal.pone.0179396 28609468 PMC5469491

[19] Cronin RS, Chelimo C, Mitchell EA, et al Survey of maternal sleep practices in late pregnancy in a multi-ethnic sample in South Auckland, New Zealand. BMC Pregnancy Childbirth 2017;17:190 10.1186/s12884-017-1378-5 28623890 PMC5474014

