# Supplementary material for: Does gentle assisted pushing or giving birth in the upright position reduce the duration of the second stage of labour? A three-arm, open-label, randomised controlled trial in South Africa
Source: BMJ Glob Health. 2018 Jun 29;3(3):e000906. doi: 10.1136/bmjgh-2018-000906 (PMC6035507; doi:10.1136/bmjgh-2018-000906)
Supplement: Supplementary data [file bmjgh-2018-000906supp001.pdf]

Hofmeyr et al. Reproductive Health (2015) 12:114
DOI 10.1186/s12978-015-0105-9

Reproductive Health

STUDY PROTOCOL Open Access

A multicentre randomized controlled trial of gentle assisted pushing in the upright posture (GAP) or upright posture alone compared with routine practice to reduce prolonged second stage of labour (the Gentle Assisted Pushing study): study protocol

G. Justus Hofmeyr[1], Mandisa Singata[1], Theresa Lawrie[2], Joshua P. Vogel[2*], Sihem Landoulsi[2], Armando H. Seuc[2] and A. Metin Gülmezoglu[2]

Abstract

Background: Fundal pressure (pushing on the upper part of the uterus in the direction of the birth canal) is often performed in routine practice, however the benefit and indications for its use are unclear and vigorous pressure is potentially harmful. There is some evidence that it may be applied routinely or to expedite delivery in some situations (e.g. fetal distress or maternal exhaustion), particularly in settings where other methods of achieving delivery (forceps, vacuum) are not available. Gentle assisted pushing (GAP) is an innovative method of applying gentle but steady pressure to the uterine fundus with the woman in an upright posture. This trial aims to evaluate the use of GAP in an upright posture, or upright posture alone, on reducing the mean time of delivery and the associated maternal and neonatal complications in women not having delivered following 15-30 min in the second stage of labour.

Methods/Design: We will conduct a multicentre, randomized, unblinded, controlled trial with three parallel arms (1:1:1). 1,145 women will be randomized at three hospitals in South Africa. Women will be eligible for inclusion if they are ≥18 years old, nulliparous, gestational age ≥ 35 weeks, have a singleton pregnancy in cephalic presentation and vaginal delivery anticipated. Women with chronic medical conditions or obstetric complications are not eligible. If eligible women are undelivered following 15-30 min in the second stage of labour, they will be randomly assigned to: 1) GAP in the upright posture, 2) upright posture only and 3) routine practice (recumbent/supine posture). The primary outcome is the mean time from randomization to complete delivery. Secondary outcomes include operative delivery, adverse neonatal outcomes, maternal adverse events and discomfort.

Discussion: This trial will establish whether upright posture and/or a controlled method of applying fundal pressure (GAP) can improve labour outcomes for women and their babies. If fundal pressure is found to have a measurable beneficial effect, this gentle approach can be promoted as a replacement for the uncontrolled methods currently in use. If it is not found to be useful, fundal pressure can be discouraged.

* Correspondence: vogeljo@who.int
[2]UNDP/UNFPA/UNICEF/WHO/World Bank Special Programme of Research, Development and Research Training in Human Reproduction (HRP), Department of Reproductive Health and Research, World Health Organization, Avenue Appia 20, Geneva, Switzerland
Full list of author information is available at the end of the article

© 2015 Hofmeyr et al. **Open Access** This article is distributed under the terms of the Creative Commons Attribution 4.0 International License (http://creativecommons.org/licenses/by/4.0/), which permits unrestricted use, distribution, and reproduction in any medium, provided you give appropriate credit to the original author(s) and the source, provide a link to the Creative Commons license, and indicate if changes were made. The Creative Commons Public Domain Dedication waiver (http://creativecommons.org/publicdomain/zero/1.0/) applies to the data made available in this article, unless otherwise stated.

Background

The first stage of labour is the period of time from the onset of intense, synchronous and co-ordinated contractions leading to full cervical dilatation. The second stage of labour is the period from full cervical dilatation to expulsion of the baby through the vagina. UK guidelines define the normal duration of the second stage of labour to be about 0.5 to 2.5 h for nulliparous women, and up to 2 h in parous women following the onset of the active second stage of labour [1]. There is little consensus as to what constitutes a prolonged second stage of labour. UK guidelines define it as more than two hours and one hour of the onset of the active second stage for nulliparous and parous women, respectively [1]. After this time, women in the UK are referred for operative vaginal birth. Poor contractions, maternal exhaustion, epidural analgesia and occipito-posterior fetal positions are common causes of prolonged second stage. These causes can be difficult to overcome without interventions such as oxytocin (to stimulate or augment uterine contractions) and assisted vaginal birth (such as forceps or vacuum).

Fundal pressure involves the application of pressure by the birth attendant to the uppermost part of a woman's uterus, directed towards the birth canal, in an attempt to assist spontaneous vaginal delivery [2]. Applying manual fundal pressure was originally described by Samuel Kristeller in the 1870s and was known as the Kristeller manoeuvre. Nowadays, although this manoeuvre is not officially taught to midwives and doctors, the use of manual fundal pressure is still performed in clinical practice, although often not documented in clinical records [3]. Applying fundal pressure under controlled conditions has been shown to increase intrauterine pressure [4]. This increase in the expulsive uterine forces could potentially assist the mother to push the baby out. There is evidence to show that fundal pressure is used in formal and informal childbirth practice, in developed and less developed countries [5–8]. However, how frequently fundal pressure is performed in routine practice and the indications for its' use in different settings is unclear. In a 2006 postpartum survey of women in the USA, it was reported that there was a 'notable minority' of births during which 'a staff member pressed on the mother's belly to help push the baby out' [7]. A survey in the Netherlands between 1994 and 1995 reported a prevalence of 4 % [8]. Two other studies in tertiary care institutions have reported the incidence of fundal pressure application to be 23-24 % [9, 10]. It may be applied routinely, in prolonged second stage of labour, for situations where delivery needs to be expedited (e.g. fetal distress) or for maternal medical conditions where prolonged pushing is contraindicated [2]. Fundal pressure is also used routinely to assist birth at Caesarean section.

The safety of this intervention has not been established [3, 11–13], and its use remains controversial [13]. Applying fundal pressure has been both recommended and condemned as a strategy for assisting birth in the management of shoulder dystocia [14, 15]. To our knowledge, the only published randomized controlled trials (RCTs) of manual fundal pressure during vaginal birth have found no benefits associated with its use, but the numbers studied were small [16, 30]. A pilot RCT of manual fundal pressure compared with standard care in 209 nulliparous women with uncomplicated pregnancies in a hospital in India found no significant reduction in the duration of the second stage of labour, but an increase in perineal injuries [16]. A 2005 review of the manoeuvre concluded that the role of fundal pressure is understudied and caution should be exercised until it is proven to be safe and effective [11]. A 2009 Cochrane review concluded that there is currently insufficient high quality evidence to draw firm conclusions about the benefits and risks of this practice [2].

Fundal pressure is usually applied manually; however, two small RCTs (conducted in hospitals in South Korea and Italy) have evaluated fundal pressure applied using two different types of inflatable belts compared with standard care in nulliparous women with uncomplicated singleton pregnancies [17, 18]. These studies reported a significant reduction in the duration of the second stage of labour and, in the study in Italy, a significant reduction in perineal trauma, CS, vacuum delivery and neonatal ICU admission.

Potential benefits and risks of fundal pressure

In well-resourced settings, a prolonged second stage of labour is usually overcome by assisted vaginal delivery or caesarean section. In lower-income countries, for the majority of births, instrumental or operative delivery methods may not be available; and timely transfer to a service with these facilities may not be feasible. In these settings, fundal pressure may be an effective way of expediting the birth of a baby, and potentially reducing the risk of neonatal morbidity and mortality associated with a prolonged and difficult birth. In addition, this intervention may be valuable for women in whom instrumental delivery is contraindicated (such as HIV infection) and for whom assistance in the second stage of labour is necessary. However, even in settings where instrumental or operative delivery is available, fundal pressure may reduce the need for their use and, thereby, reduce the risk of associated third-degree tears or surgery. The potential benefits, therefore, have implications for obstetric care at all levels.

Fundal pressure manoeuvres of variable force and direction are commonly used by birth attendants with potentially harmful effects. Maternal complications ascribed to

these ‘conventional’ fundal pressure manoeuvres include perineal tears, uterine rupture, uterine inversion, pain, hypotension, respiratory distress, abdominal bruising, fractured ribs, and liver rupture [19–26]. Excessive fundal pressure has also been cited as a predisposing factor for amniotic fluid embolism [27]. In the neonate, fundal pressure has been implicated as a possible cause of fractures and brain damage [28, 29]. The theoretical basis for the latter is that applying mechanical forces to the uterus to increase intrauterine pressure could cause a concomitant increase in fetal intracranial pressure. This, in turn, could lead to a decrease in fetal cerebral blood flow and subsequent cerebral handicap in the infant. The same mechanism has been postulated to occur in cases of cerebral palsy following uterine tetany or forceps delivery. In addition, if used in the presence of cephalo-pelvic disproportion, the risk of maternal and neonatal injury would be high.

There is also a theoretical possibility that fundal pressure may cause maternal-fetal transfusion of blood, increasing the risk of viral transmission such as HIV. However, in a randomized trial of fundal pressure versus no fundal pressure at caesarean section, no evidence of maternal-fetal transfusion was found [30].

The lack of a standardised method of application is also contributing to differences of opinion regarding the utility of fundal pressure. Although it has been described as being ‘applied gently with one hand on the uterine fundus at a 30° to 45° angle to the maternal spine in the direction of the pelvis’ [13], in a longitudinal direction, it is difficult to quantify or control the amount of force used. Fundal pressure may vary from the gentle pressure described above, to vigorous force applied with both hands or elbows using the attendant’s whole weight. A tendency to use rapid thrusting movements may cause sharp rises in intrauterine pressure and increase the risk of maternal and neonatal injury. The gentle assisted pushing (GAP) method described in this protocol is designed to eliminate the possibility of excessive use of force, by positioning the attendant behind the women who is in an upright position, so that only the pressure applied with her hands can be used.

The role of maternal posture

In the 1800s, it was noted that women would give birth in upright positions using posts, hammocks or furniture, or that women might kneel, crouch, or squat using bricks, stones, or a birthing stool [31]. Upright positions adopted during the first stage of labour have been shown in a systematic review to reduce the duration of labour by about one hour [32]. Upright postures may enhance progress in the second stage of labour and reduce operative delivery, however the limited evidence available is inconclusive. A systematic review of maternal position in the second stage of labour revealed several benefits due to upright posture (e.g. fewer instrumental deliveries, fewer abnormal fetal heart rate patterns, a trend to shorter second stage) however, it also revealed a possible association with more blood loss [33]. In this review, it was suggested that blood loss may have been more accurately estimated in the women allocated to upright birth in a specially designed birth chair in which blood loss was collected in a receptacle. Assuming an upright kneeling or squatting position for the second stage of labour may facilitate vaginal delivery, by enabling the mother to bear down more efficiently with the alignment of physiological and gravitational forces, as well as tilting the pelvis to a more favourable (less oblique) orientation to the direction of the uterine and bearing down forces. However, in practice the second stage of labour is often performed in recumbent or semi-recumbent positions, which some claim enables birth attendants to better monitor and gain access to the baby [31].

Pilot study

We conducted a pilot study in the form of a RCT, to determine the feasibility of a larger RCT to evaluate this new method of gentle fundal pressure called “gentle assisted pushing” (GAP), which was performed with the woman in an upright posture. The pilot study protocol was approved by the Ethics Committee, East London Hospital Complex, and the Committee for Research on Human Subjects, University of the Witwatersrand.

This two-armed RCT recruited 120 healthy, nulliparous women with a singleton pregnancy in cephalic presentation at ≥35 weeks who consented to participate. Women undelivered by 15 min of bearing down (second stage) were randomized (1:1) to 1) gentle assisted pushing in an upright posture, or 2) upright posture only. The attendants were two research midwives who were taught the fundal pressure technique by the lead investigator. During contractions, steady firm fundal pressure was applied using the palms of both hands in the direction of the pelvis using only the strength of her forearms. Steady, sustained pressure was maintained for the full duration of each contraction or 30 s, whichever was shorter. Forceful or rapid pressure and the use of body weight to apply pressure were avoided. The results of this unpublished pilot study showed a non-significant trend towards reduce rate of operative delivery. From this pilot, it was concluded that the GAP method was clinically feasible and that the results justified a larger trial to study the effectiveness and safety of this intervention, and its relationship to posture, in the second stage of labour.

This larger trial will add to the body of knowledge on upright posture versus recumbent posture. By additionally using gentle assisted pressure combined with the upright posture, the expulsive forces acting on the

uterus should be optimised. To differentiate possible effects of gentle assisted pushing and posture, and to supplement the evidence base on the effectiveness of upright posture, a three-armed comparison will be used.

Study objectives

This three-arm trial aims to evaluate the use of gentle assisted pushing in an upright posture (GAP). We will determine whether:

1. GAP is associated with reduced duration of second stage of labour, and improved neonatal and maternal outcomes, compared with current routine practice (typically recumbent/supine posture).
2. Upright posture alone is associated with a reduced duration of second stage of labour, and improved neonatal and maternal outcomes, compared with current routine practice (typically recumbent/supine posture).
3. GAP (gentle fundal pressure in an upright posture) is associated with reduced duration of second stage of labour, and improved neonatal and maternal outcomes, compared with upright posture alone.

Methods

Study design

The GAP study is a multicentre, randomized, controlled, unblinded, clinical trial with three parallel arms (1:1:1). Please see Fig. 1 for a graphical outline of the study.

Study setting

The study will be conducted in four sites at three maternity facilities in South Africa, namely:

- Frere Hospital, East London – a tertiary-level hospital with approximately 800 beds in total, with an estimated 570 deliveries per month. There are two study sites at Frere Hospital:
  - Dancan Village Day Hospital - Midwife-led obstetric unit
  - Frere labour ward - Obstetrician-led obstetric unit
- Cecilia Makiwane Hospital, East London – a secondary-level hospital with approximately 700 beds in total, with an estimated 400 deliveries per month
- Butterworth Hospital – a district hospital with approximately 400 beds in total, with an estimated 400 deliveries per month

Study participants

All women attending participating hospitals for delivery (who meet the inclusion criteria) during the study period will be approached for participation in this study.

The eligibility criteria are:

- Equal to or greater than 18 years old;
- Nulliparous women;
- Gestational age ≥ 35 weeks;
- Singleton pregnancy;

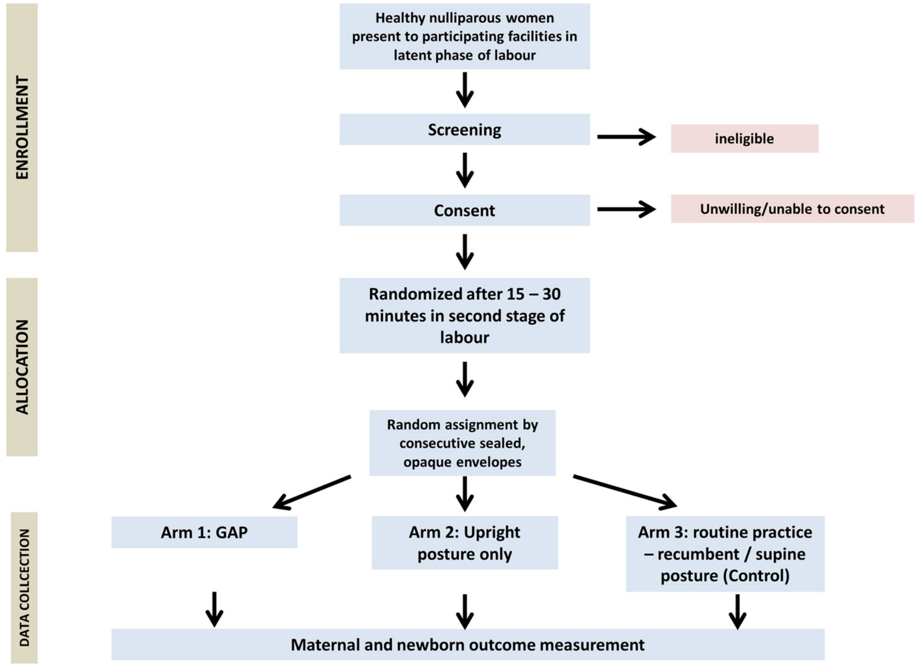

Fig. 1 Flow chart for the GAP study

- Vaginal delivery anticipated;
- Cephalic fetal presentation;
- Baby's heartbeat detected;
- No chronic medical conditions, including heart disease, epilepsy, hypertension, diabetes mellitus and renal disease;
- No obstetric complications, including hypertensive disorders of pregnancy, cephalo-pelvic disproportion, antepartum haemorrhage, intra-uterine growth restriction, fetal distress, intra-amniotic infection; and
- Willing and able to provide consent

Women whose labours are induced or augmented, or women with HIV, are not excluded from this trial. However, this information will be captured in the trial forms.

Recruitment and screening procedures

In participating facilities, nulliparous women ≥18 years old will be approached with information about the trial. Women who are in latent phase of labour and who meet the eligibility criteria will be fully counselled and informed about the trial. The following guidelines will be used for screening:

- The woman should be sufficiently comfortable to make an informed choice.
- Information should be provided in her home language.
- She will be given an information sheet and offered time to reflect and ask questions, or to consult with family members.
- If she agrees, she will sign the informed consent form
- Illiterate participants will have the consent form read to them.
- Verbal consent will be witnessed by an independent signatory.
- Following receipt of the signed consent form, baseline demographic and clinical data will be recorded on the screening form.
- Screened women will be given a screening number, including women who decline to take part and those who are not eligible. All screening forms will be kept by the study team.

Sampling and allocation

Recruited women who reach second stage of labour without major complications will be randomized. The randomization sequence will be generated by the WHO/RHR Statistics and Informatics (SIS) team. This will be computer-generated in balanced blocks of variable size, in a ratio of 1:1:1 for the three study arms. Details of the group allocation will be concealed on cards inside a sequentially-numbered series of sealed, opaque envelopes. These will be prepared by the SIS team, separately for each study site. Allocation will be performed by the research assistant, by opening the next numbered envelope in the series. The assignment schedule will be stored at WHO, and will not be accessible to the on-site research teams.

Study interventions

Participants will be allocated to one of the following intervention groups:

- Arm 1: GAP (gentle fundal pressure in an upright posture)
- Arm 2: Upright posture only
- Arm 3: Routine practice (typically recumbent/supine posture)

Labour ward staff at the study sites will be trained in these study procedures before the start of recruitment.

Arm 1: GAP (gentle fundal pressure in an upright posture)

The principle of the procedure is for the birth attendant (a midwife or doctor) to apply steady (firm yet gentle) manual pressure on the uterine fundus in the direction of the pelvis, for the full duration of each uterine contraction, but not exceeding 30 s. Forceful or rapid pressure is to be avoided.

Gentle assisted pressure will be applied by attendants who have been trained in the procedure, via a video teaching aid and practical demonstration by the study team. These training materials and methods will be standardized to ensure comparability of training and GAP administration across study sites. The woman will be assisted to assume an upright kneeling or squatting posture on the bed. The trained birth attendant will kneel behind her on the bed or stand behind her with the woman positioned at right angles to the length of the bed and back close to the side of the bed. The trained birth attendant will wrap her arms around the woman passing below her axillae, and place both open palms, overlapping, on the fundus of her uterus. Steady pressure in the long axis of the uterus will be applied only during contractions. The duration of pressure will be limited to 30 s, with a minimum of 30 s rest before the next pressure.

The position of the trained birth attendant in relation to the mother ensures that excessive force using the assistant's weight cannot be used, as occurs with conventional fundal pressure when the woman is supine and the trained birth attendant is standing alongside her. The upright posture is integral to this intervention. During training, a manikin will be used with a small air-filled bladder between the trained birth attendant's hands and the manikin

abdomen, connected to a beaumanometer so that the amount of pressure can be monitored.

Arm 2: Upright posture

Participating women will be encouraged to assume an upright crouching or kneeling position during the second stage of labour. When the baby's head is 'crowning', the trained birth attendant may choose to move the woman to a recumbent position for the birth.

Arm 3: Routine practice (recumbent/supine posture only)

The current posture as practiced in the participating sites will be used.

In all intervention arms, the trained birth attendant encourages the women to bear down. All other delivery procedures, such as fetal heart monitoring and routine oxytocin after the birth, are performed according to the usual hospital routine and will not differ between the groups.

For Arm 3 (routine practice), if the woman remains undelivered 30 min after randomization, fundal pressure or changes in posture may be considered necessary by the birth attendant according to routine local practice. Additional procedures or treatments performed at the discretion of the attendant will then be recorded in the study form.

At any stage of the labour, routine procedures such as change in posture, oxytocin administration, forceps or vacuum delivery or caesarean section will be available as considered indicated by the responsible clinician. If forceps, vacuum delivery or caesarean section is required, GAP and/or upright only posture will be stopped.

Electronic fetal heart rate monitoring is not routinely used in the study institutions in uncomplicated pregnancies; therefore, this equipment is unlikely to interfere with the administration of the interventions in this study. Auscultation of the fetal heart rate will be performed after each contraction following randomization. If electronic monitoring is considered necessary by the birth attendant, the GAP procedure will not interfere with monitoring as the monitor is positioned on the anterior aspect of the uterus while pressure is applied to the fundal aspect.

Study outcomes

The primary outcome is defined as mean time (minutes) from randomization to delivery.

Secondary outcomes include the following:

- Delivery outcomes:
  - No spontaneous delivery within 15 min of randomization;
  - Operative delivery (vacuum, forceps or caesarean section); and
  - Episiotomy or 2nd/3rd degree tears.
- Neonatal outcomes:
  - Cord blood pH < 7.2;
  - 5-min Apgar score <7;
  - Neonatal injury;
  - Neonatal encephalopathy;
  - Admission to neonatal high care nursery for ≥24 h; and
  - Neonatal death.

Mothers will also be asked to grade their discomfort experienced during the second stage of labour. All adverse events and serious adverse events will also be recorded.

Safety considerations

No drugs are being tested in this study. We are testing interventions that are not taught or sanctioned by teaching institutions in South Africa or elsewhere due to lack of evidence, but practiced to varying degrees of frequency. WHO does not currently recommend this procedure. The GAP method of applying fundal pressure has been specifically developed to avoid the commonly used, more vigorous form of fundal pressure. Therefore, interventions which are practiced to some extent (fundal pressure and posture) but for which there is little supporting evidence are being tested by the study. We do not anticipate any specific safety concerns over and above those usually anticipated in the second stage of labour.

However, some women may find specific birth positions and/or gentle fundal pressure uncomfortable. The research staff will assist and support women to use the allocated study position. Women may benefit from the additional support. However, should a woman choose to change birth position, or withdraw from the study for any reason, it will not affect the medical care to which she is entitled.

Adverse events

An adverse event is any untoward medical occurrence in a patient or clinical investigation subject and which does not necessarily have a causal relationship with this treatment. An adverse event can therefore be any unfavourable and unintended sign (including an abnormal laboratory finding), symptom, or disease temporally associated with the use of a medicinal (investigational) product, whether or not related to the medicinal (investigational) product.

Any adverse event that requires treatment will be reported an Adverse Event (AE) form and the principal investigator immediately alerted.

The serious adverse event form will be completed in the following circumstances:

- The woman has been transferred to another department or hospital in relation to an adverse event after enrolment in the trial.
- An adverse event has caused prolonged hospital stay.
- The woman has been re-admitted to the hospital for further care of an adverse event that has occurred while she was in the trial.
- The adverse event has caused permanent loss of function of a system or organ.
- The adverse event is considered to be life threatening.
- The adverse event has resulted in death.

Reporting of AEs and SAEs will be done using an adverse event (AE) form and serious adverse event (SAE) form. These forms will be completed by data collectors and the principal investigator informed as soon as possible. The WHO project manager will be notified of the AE or SAE within 24 h, and the DSMC will be notified of SAEs within 24 h (accelerated reporting).

Criteria for discontinuation

Criteria for discontinuation of a participant

Participants who no longer wish to remain in the study will be discontinued.

Criteria for discontinuation of the study

An interim analysis by the statistical team is planned after 50 % of women have completed the study. These results will be reviewed by the Data Safety and Monitoring Committee (DSMC). All adverse events and severe adverse events will be provided to the DSMC on an expedited basis. If the Committee feels the data confirms significant harm or benefit attributed to one of the study arms and recommends discontinuation of the arm or the study, enrolment will be stopped accordingly.

Follow-up procedures

Delivery details of screened, consenting women who are not enrolled/randomized (e.g. who deliver before enrolment by caesarean section or vaginal delivery) will be recorded on the relevant screening form.

Participating women and their neonates must be followed up until discharge from the maternity and neonatal units. Therefore, we do not anticipate any loss to follow-up. The case report forms are available upon request.

For quality control, in a subset of cases a mobile phone or camera-based video and sound recorder with recorded time display may be set up behind the woman prior to random allocation. The recorder will be positioned so that the mother's identity is not seen. The researcher's recorded voice must document the time at which the randomization envelope is opened, the posture of the woman, the use of fundal pressure, and the time of delivery of the baby. The times will be transcribed from the time sheet and/or the recording to the paper CRF.

Recordings will only be used for research purposes; to assess quality of recording of timing of events. These recordings will be kept securely with other trial documentation in the research office and be accessible only to research staff. At the end of the study all recordings will be destroyed.

Data management and quality assurance

Both the protocol and the trial report will include requirements laid out in the CONSORT statemen[34 - see reference on CONSORT]t. The protocol is registered with the Pan African Clinical Trials Registry (PACTR201502001034448). The first version of the statistical analysis plan will be finalised before recruitment begins. Participating research staff will be trained to correctly introduce the study to potential participants, administer the consent and correctly complete all study forms as per the manual of operations and for entering data in the web-based system. Each trial site will pilot the trial procedures, including pilot testing the forms, before recruitment. Refresher courses will be conducted as considered necessary.

Randomization will take place when the woman's name and hospital number is written in the randomization register. Good Clinical Practice (GCP) procedures will be followed. Data will be collected by research staff onto paper CRFs.

Quality control will be performed on-site: a member of each research team will be responsible for data entry and query management, and ensuring that the CRFs are correctly filled out. Completed CRFs will be randomly checked by another member against the original patient records for correctness. Any discrepancies will be immediately addressed.

Data entry

The data will be entered in OpenClinica™, a web-based, fully GCP-compliant data management system. The data analyst at WHO Geneva will develop the data management system, provide hosting, data back-ups and maintenance of the system.

A validation system will be built into the data management system to ensure consistency, accuracy and completeness of the data collected (range checks, consistency checks and skips). Double data entry will be performed and queries will be resolved as much as possible before discharge of the woman from hospital. First and second data entry will be done as soon as the case report forms are completed.

It is unlikely that serious side-effects of the intervention will occur. Adverse events and serious adverse events, whether thought to be related to the trial intervention or not, will be recorded by the researchers on the adverse events CRFs which will be submitted to the DSMC and the relevant ethics committees.

Data protection procedures

The following measures will be taken to ensure participant confidentiality:

- Trial data for each participant will be identified by a unique ID number, not the participant's name
- The trial register of names and trial ID numbers will be kept separate from the CRFs
- Trial documents will be kept securely under lock and key in the research offices and will not be accessible, other than to the researchers
- Data will be entered by trial ID number in the password-protected data management system to which only trial staff will have access
- The trial report will not contain the names of any participants
- After completion of the trial, the trial's documents will be kept secured for five years

Statistical methods

The sample size has been calculated to be powered to detect a reduction in the primary outcome of mean time (minutes) from randomization to delivery of > =3 min. A reduction of > =3 min was used due to the findings of the study by Api et al (2009) [31], that reported a time from randomization to delivery of 23.1 min (SD 12.2 min) in the control group ($N = 56$), and 18.6 min (SD 9.5 min) in the fundal pressure group ($N = 34$). We assumed that upright posture alone would likely lie between these two (i.e. approximately 20.0 min). Using the Bonferroni rule to control for multiplicity, alpha is divided by the number of comparisons ($0.05/3 \approx 0.02$). We assumed a power of 90 % and alpha of 95 %. The study therefore requires 347 women in each arm, i.e. 1,041 women for all three arms. To allow for 10 % noncompliance, we aim to recruit 382 in each arm. The final sample size for the whole study is 1,145 women.

Data analysis

Data will be generated from the on-line data management system and analysed in SPSS. The primary analysis will be based on all subjects with outcome data available. Any participants with protocol violations, including women who no longer wish to receive their allocated intervention but are prepared to have data collected, will be analysed in the group to which they were allocated (intention-to-treat). Data from subjects who withdraw their consent will be excluded from the analysis and considered as lost to follow-up.

Baseline characteristics will be compared between groups to confirm the effectiveness of the randomization process in creating similar groups. Numbers and baseline characteristics of women lost to follow-up will also be compared between study groups to detect any imbalances.

Most study outcomes are categorical variables. For these types of variables, the number of subjects, number of missing values and percentages will be reported. Categorical variables will be compared between study arms as risk ratios with 95 % confidence intervals.

For continuous variables, e.g. time to delivery, the number of subjects, and number of missing values, minimum, maximum, means and standard deviations will be reported. Appropriate transformation of non-normal or skewed measurement will be performed. Means will be compared using t-tests for two independent samples, and 95 % confidence intervals will be calculated. If transformations do not generate a distribution approaching normality, medians and interquartile range (IQR) will be reported. The primary outcome of the study is "mean time (minutes) from randomization to delivery". The survival technique log-rank test will also be used to compare the survival rates of the three groups, pairwise. Results will be reported according to CONSORT guidelines [22].

Discussion

Expected outcomes of this study

The current widespread use of uncontrolled fundal pressure is an important public health and human rights issue, which this trial will seek to address. The study will establish whether upright posture and/or a controlled method of applying fundal pressure (GAP) can improve labour outcomes for women and/or their babies. If fundal pressure is found to have a measurable beneficial effect in this study, this gentle approach can be promoted as a replacement for the uncontrolled methods currently in use. If it is not found to be useful in this study, fundal pressure can be discouraged.

Dissemination

The investigators are committed to the widespread dissemination of the findings of this study. We will seek to publish the results in a peer-reviewed journal, in addition to making the results available to the country Department of Health, to inform labour care policy and training. Study data will also be incorporated into our review of fundal pressure in the Cochrane Library, which is recognised as a leading source of evidence-based information. The review will appear in the WHO Reproductive Health Library,

which is freely available on the internet in developing countries, and in several languages.

Anticipated problems and solutions

The main potential problem that we anticipate may be a slow rate of recruitment. To minimize this possibility, dedicated research staff will be appointed at the recruiting sites. In addition, recruitment will be closely monitored and, if inadequate, consideration will be given to including additional sites or extending the duration of the study. The trial procedures are straightforward and have been shown in a pilot study to be feasible. As posture during labour is a potential confounding factor for studies of fundal pressure, we have eliminated the potential for this variable to confound results by designing a three-arm trial.

Project management

This project will be managed by the UNDP/UNFPA/UNICEF/WHO/World Bank Special Programme of Research, Development and Research Training in Human Reproduction (HRP), Department of Reproductive Health and Research, World Health Organization, Geneva, Switzerland. In South Africa, the country principal investigators will establish the research teams that will implement the research activities. The study coordinating unit in Geneva will conduct site visits before and during the implementation of the study to contribute to study site selection, training workshops and assessment of adherence to study protocols. There will be continuous communication between country research teams and study coordinating unit at the WHO. Regular contact will be made to ensure that the timeline are followed and problems solved without delay.

A Data Safety Monitoring Committee (DSMC) will be established to safeguard the interests of the trial's participants, potential participants, investigators and sponsors, to assess the safety and efficacy of the trial's intervention according to adverse event data available while the trial is ongoing, and all other data available at a predefined schedule, to monitor the trial's overall conduct and quality, and protect its validity and credibility and to make recommendations concerning continuation or termination of study or any other modification necessary based on the observed effects of the intervention.

Ethical considerations

The main ethical consideration relates to the timing of recruitment. The WHO RHR and the Effective Care Research Unit have extensive experience in recruitment of women for interventions around the time of childbirth. These trials have shown that it is feasible to recruit women in early labour, and that it is acceptable to women.

All women will receive information about the trial in their language of choice, conforming to ethical requirements for research involving human subjects. The language is non-technical and easily understood. Participants will be given time to reflect on the information. After signing the informed consent form, participants will be free to withdraw from the trial at any stage without loss of benefits. If a woman is illiterate, an impartial witness will be present during the entire informed consent reading and discussion. The impartial witness will also sign and date the ICF, along with the individual who performed the informed consent discussion. The telephone numbers of investigators will be made available participants in the event that they require further information or assistance.

There will be no payment for participation. Only compensation for time spent completing trial procedures as approved or requested by local ethics committees to be appropriate and non-coercive will be considered.

The study protocol (WHO Study A65866) was reviewed and approved by the WHO Research Project Review Panel (RP2) and the WHO Ethics Review Committee. Ethics The University of the Witwatersrand Committee for Research on Human Subjects (HREC) has approved this study protocol (Ethics clearance certificate no. M131054).

Abbreviations

AE: Adverse event; ASCII: American standard code for information interchange; CONSORT: Consolidated standards of reporting trials; CRF: Case report forms; DSMC: Data safety and monitoring committee; ECRU: Effective care research unit; GAP: Gentle assisted pushing; GCP: Good clinical practice; ICF: Informed consent form; ICRHK: International centre for reproductive health Kenya; ID: Identification; PI: Principal investigator; SAE: Serious adverse event; SAS: Statistical analysis software; SIS: Statistics and informatics services; TSC: Trial steering committee; WHO: World health organization.

Competing interests

The authors declare that they have no competing interest.

Authors' contributions

The concept for this study was developed by GJH and AMG. The protocol was drafted by TL, MS and GJH, with substantial inputs from JPV, AS, SL and AMG. JPV developed this manuscript based on the final protocol. All named authors reviewed and approved the manuscript.

Acknowledgments

We would like to thank Mariana Widmer and Maria Rodriguez for their technical input in the development of the study forms.

Author details

[1]Department of Obstetrics and Gynaecology at East London Hospital Complex (ELHC), Effective Care Research Unit (ECRU), and Eastern Cape Department of Health, University of the Witwatersrand, University of Fort Hare, East London, South Africa. [2]UNDP/UNFPA/UNICEF/WHO/World Bank Special Programme of Research, Development and Research Training in Human Reproduction (HRP), Department of Reproductive Health and Research, World Health Organization, Avenue Appia 20, Geneva, Switzerland.

Received: 27 November 2015 Accepted: 4 December 2015
Published online: 16 December 2015

References

1. National Collaborating Centre for Women's and Children's Health (UK). Intrapartum care: care of healthy women and their babies during childbirth. London: RCOG Press; 2007. (NICE Clinical Guidelines, No. 55.) 8, Normal

labour: second stage. Available from: http://www.ncbi.nlm.nih.gov/books/NBK49393/.
2. Verheijen EC, Raven JH, Hofmeyr GJ. Fundal pressure during the second stage of labour (Review). Cochrane Database Syst Rev. 2009 (4). Art. No.: CD006067. http://www.ncbi.nlm.nih.gov/pubmed/19821352
3. Kline-Kaye V, Miller-Slade D. The use of fundal pressure during the second stage of labor. J Obstet Gynecol Neonatal Nurs. 1990;19:511–7.
4. Buhimschi CS, Buhimschi IA, Malinow AM, Kopelman JN, Weiner CP. The effect of fundal pressure manoeuvre on intrauterine pressure in the second stage of labour. BJOG. 2002;109(5):520–6.
5. Miller S, Cordero M, Coleman AL, Figuero J, Brito-Anderson S, Dabagh R, et al. Quality of care in institutionalized deliveries: the paradox of the Dominican Republic. Int J Gynecol Obstet. 2003;82(1):89–103.
6. Goldman N, Glei D. Evaluation of midwifery care: results from a survey in rural Guatemala. Soc Sci Med. 2003;56(4):685–700.
7. Declercq ER, Sakala C, Corry MP, Applebaum S. New York: childbirth connection. Listening to mothers II: report of the second national U.S. Survey of women's childbearing experiences. J Perinat Educ. 2007;Fall; 16(4):9–14.
8. de Leeuw JW, Vierhout ME, Struijk PC, Auwerda HJ, Bac DJ, Wallenburg HC. Anal sphincter damage after vaginal delivery: relationship of anal endosonography and manometry to anorectal complaints. Disease of Colon and Rectum. 2002;45(8):1004–10.
9. Schulz-Lobmeyr I, Zeisler H, Pateisky N, Husslein P, Joura EA. Fundal pressure during the second stage of labor: a prospective pilot study [Die kristeller–technik: eine prospective untersuchung]. Geburtshilfe Frauenheilkd. 1999;59:558–61.
10. Moiety FM, Azzam AZ. Fundal pressure during the second stage of labor in a tertiary obstetric center: A prospective analysis. J Obstet Gynaecol Res. 2014. doi: 10.1111/jog.12284. [Epub ahead of print]
11. Merhi ZO, Awonuga AO. The role of uterine fundal pressure in the management of the second stage of labor: a reappraisal. Obstet Gynecol Surv. 2005;60:599–603.
12. Goodburn EA, Gazi R, Chowdhury M. Beliefs and practices regarding delivery and postpartum maternal morbidity in rural Bangladesh. Stud Fam Plann. 1995;26:22–32.
13. Simpson KR, Knox GE. Fundal pressure during the second stage of labor. MCN Am J Matern Child Nurs. 2001;26:64–70. quiz 71.
14. Penney DS, Perlis DW. Shoulder dystocia: when to use suprapubic or fundal pressure. MCN Am J Matern Child Nurs. 1992;17:34–6.
15. Gross SJ, Shime J, Farine D. Shoulder dystocia: predictors and outcome. A five-year review. Am J Obstet Gynecol. 1987;156:334–6.
16. Mahendru R. Shortening the second stage of labor? J Turk Ger Gynecol Assoc. 2010;11(2):95–8.
17. Kim JW, Kim YH, Cho HY, Shin HY, Shin JC, Choi SK, et al. The effect of inflatable obstetric belts in nulliparous pregnant women receiving patient-controlled epidural analgesia during the second stage of labor. J Matern Fetal Neonatal Med. 2013;26(16):1623–7.
18. Acanfora L, Rampon M, Filippeschi M, Marchi M, Montisci M, Viel G, et al. An inflatable ergonomic 3-chamber fundal pressure belt to assist vaginal delivery. Int J Gynaecol Obstet. 2013;120(1):78–81.
19. Zetterstrom J, Lopez A, Anzen B, Norman M, Holmstrom B, Mellgren A. Anal sphincter tears at vaginal delivery: risk factors and clinical outcome of primary repair. Obstet Gynecol. 1999;94:21–8.
20. Cosner KR. Use of fundal pressure during second-stage labor. A pilot study. J Nurse Midwifery. 1996;41:334–7.
21. Matsuo K, Shiki Y, Yamasaki M, Shimoya K. Use of uterine fundal pressure manoeuver at vaginal delivery and risk of severe perineal laceration. Arch Gynecol Obstet. 2009;280:781–6.
22. Matsubara S, Mita F, Kikkawa I, Suzuki M. Maternal rib fracture after manual uterine fundal pressure. Rural Remote Health. 2012;12:2062.
23. Wei SC, Chen CP. Uterine rupture due to traumatic assisted fundal pressure. Taiwan J Obstet Gynecol. 2006;45:170–2.
24. Pan HS, Huang LW, Hwang JL, Lee CY, Tsai YL, Cheng WC. Uterine rupture in an unscarred uterus after application of fundal pressure. A case report. J Reprod Med. 2002;47:1044–6.
25. Van Geenderhuysen C, Souida A. Uterine rupture of pregnant uterus: study of a continuous series of 63 cases at the referral maternity of Niamey (Niger). Med Trop (Mars). 2002;62:615–8.
26. Lee WK, Baggish MS, Lashgari M. Acute inversion of the uterus. Obstet Gynecol. 1978;51:144–7.
27. Högberg U, Joelsson I. Amniotic fluid embolism in Sweden, 1951-1980. Gynecol Obstet Invest. 1985;20(3):130–7.
28. David TA. Was fundal pressure by nurses during birth cause of brain damage? Case on point: Martinez v. Northridge Hospital Medical Ctr., 2003 WL 22234957-CA. Nurs Law Regan Rep. 2003;44:2.
29. Amiel-Tison C. Cerebral handicap in full-term newborns related to late pregnancy and/or labor. Eur J Obstet Gynecol Reprod Biol. 1988;28:157–63.
30. Owens M, Bhullar A, Carlan SJ, O'Brien WF, Hirano K. Effect of fundal pressure on maternal to fetal micro-transfusion at the time of cesarean delivery. J Obstet Gynaecol Res. 2003;29:152–6.
31. Api O, Balcin ME, Ugurel V, Api M, Turan C, Unal O. The effect of uterine fundal pressure on the duration of the second stage of labor: a randomized controlled trial. Acta Obstet Gynecol Scand. 2009;88:320–4.
32. Gupta JK, Nikodem C. Maternal posture in labour. Eur J Obstet Gynecol Reprod Biol. 2000;92(2):273–7.
33. Gupta JK, Hofmeyr GJ, Shehmar M. Position in the second stage of labour for women without epidural anaesthesia. Cochrane Database of Syst Rev. 2012 (5). Art. No.: CD002006. 10.1002/14651858.CD002006.pub3. http://www.ncbi.nlm.nih.gov/pubmed/22592681
34. Schulz KF, Altman DG, Moher D, for the CONSORT Group. CONSORT 2010 statement: updated guidelines for reporting parallel group randomised trials. Ann Int Med. 2010;152. Epub 24 March.

Submit your next manuscript to BioMed Central and we will help you at every step:

- We accept pre-submission inquiries
- Our selector tool helps you to find the most relevant journal
- We provide round the clock customer support
- Convenient online submission
- Thorough peer review
- Inclusion in PubMed and all major indexing services
- Maximum visibility for your research

Submit your manuscript at www.biomedcentral.com/submit

BioMed Central
