# Supplementary figures and images for: Does gentle assisted pushing or giving birth in the upright position reduce the duration of the second stage of labour? A three-arm, open-label, randomised controlled trial in South Africa
Source: BMJ Glob Health. 2018 Jun 29;3(3):e000906. doi: 10.1136/bmjgh-2018-000906 (PMC6035507; doi:10.1136/bmjgh-2018-000906)

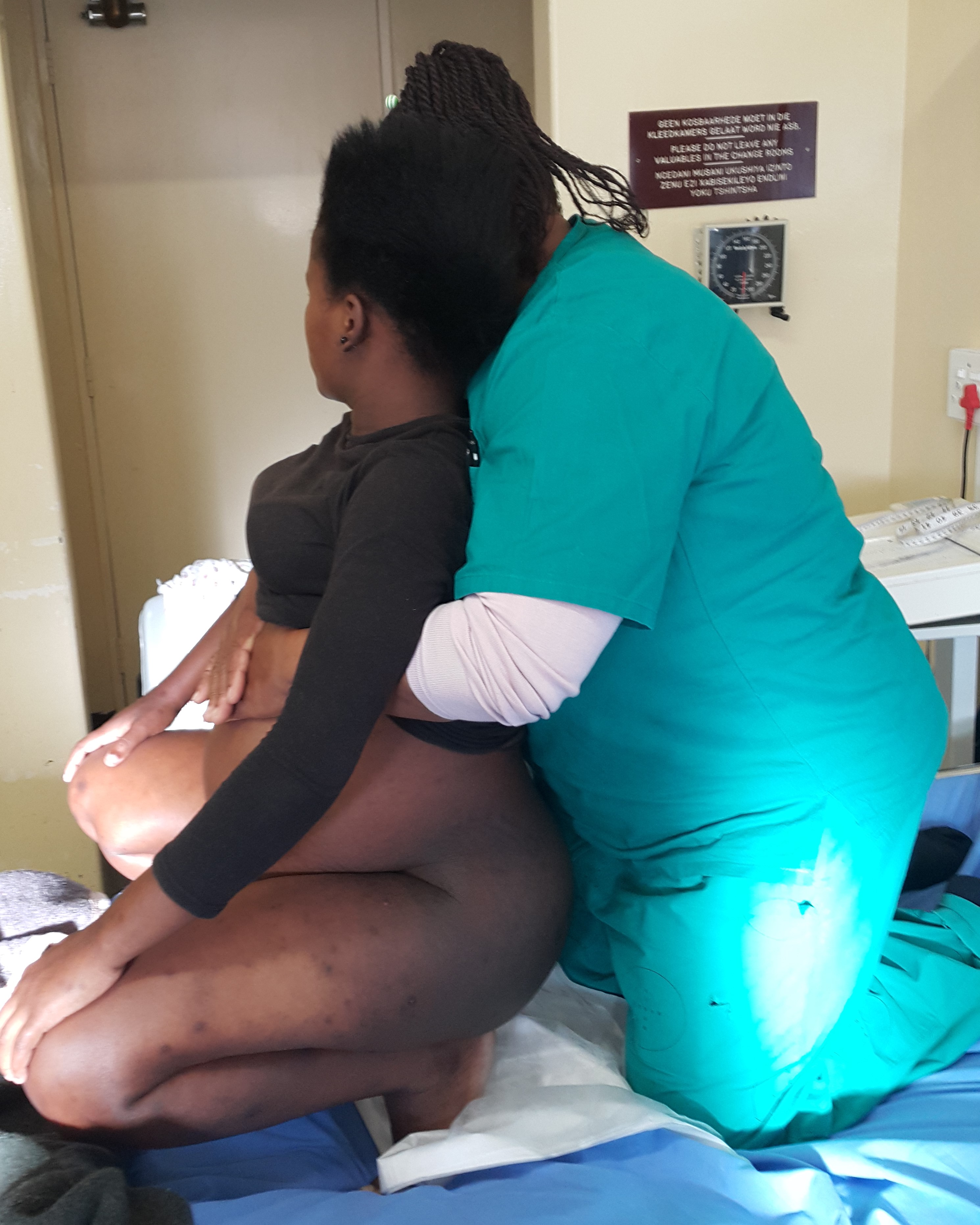

Supplement: Supplementary data [file bmjgh-2018-000906supp003.jpg]
