# Supplementary material for: Does gentle assisted pushing or giving birth in the upright position reduce the duration of the second stage of labour? A three-arm, open-label, randomised controlled trial in South Africa
Source: BMJ Glob Health. 2018 Jun 29;3(3):e000906. doi: 10.1136/bmjgh-2018-000906 (PMC6035507; doi:10.1136/bmjgh-2018-000906)
Supplement: Supplementary data [file bmjgh-2018-000906supp004.pdf]

SUPPLEMENTARY APPENDIX

SUPPLEMENTARY FIGURE 1. Time to birth by any mode, Kaplan-Meier curve

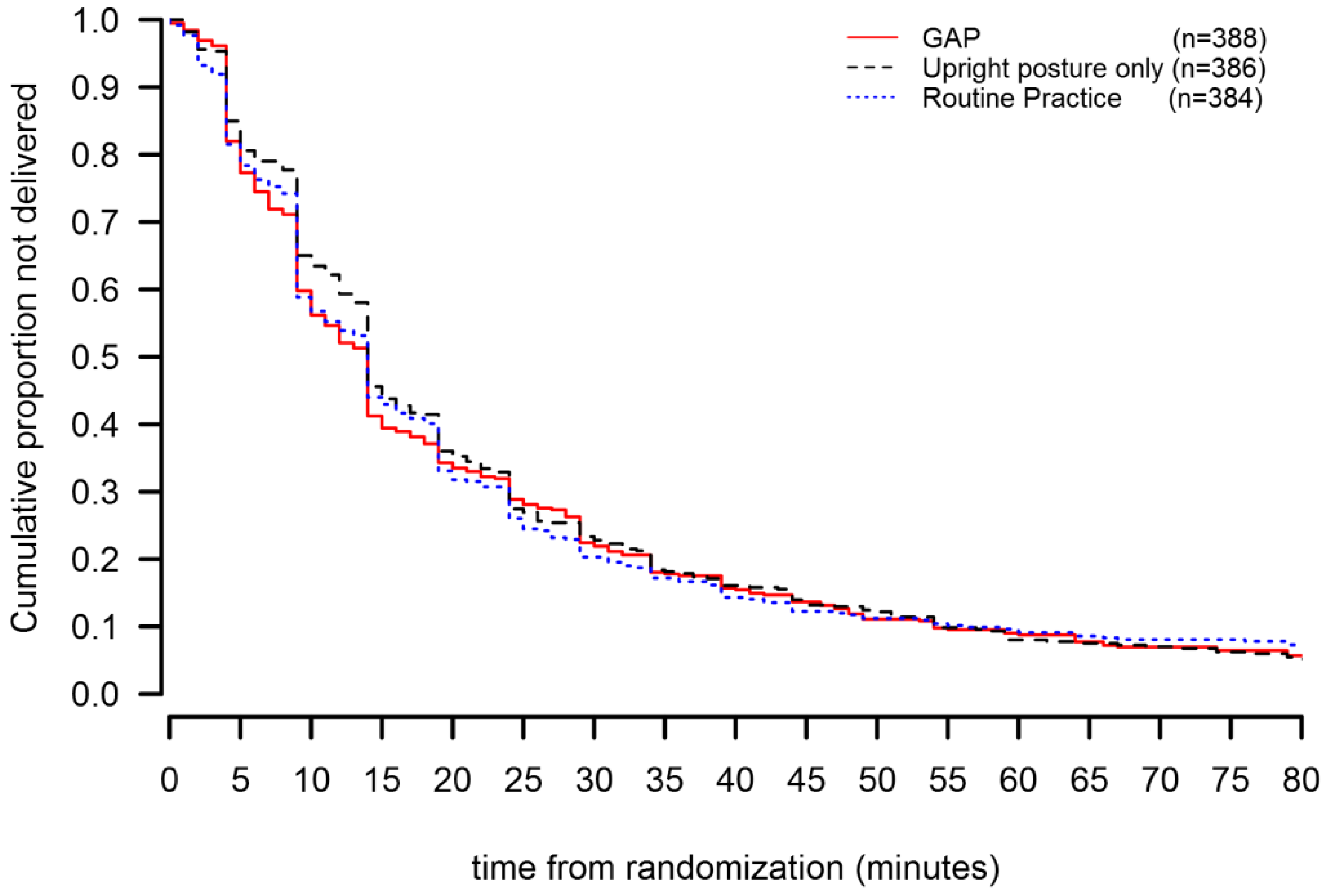

Supplementary Table 1. Adjusted difference in mean log duration across three arms

Adjusted Difference in Mean Log duration[a] (Wald 95% confidence interval)					
**GAP vs Routine practice**	**Wald Chi-square p-value**	**Upright versus routine practice**	**Wald Chi-square p-value**	**GAP vs upright**	**Wald Chi-square p-value**
0.04 (-0.14, 0.20)	0.69	0.14 (-0.03, 0.31)	0.108	-0.11 (-0.28, 0.06)	0.21

[a] Adjusted for centre

Supplementary Table 2. Kaplan-Meier proportion of women delivered (any mode) from randomization to birth (in minutes)

Duration to birth (minutes)	GAP		Upright position only		Routine practice	
	At risk /events (cum)	Proportion delivered (95% CI)	At risk /events (cum)	Proportion delivered (95% CI)	At risk /events (cum)	Proportion delivered (95% CI)
0	388/2	0.5 (0.1, 2.0)	386/0	0.00	384/3	0.8 (0.3, 2.4)
5	318/88	22.7 (18.8, 27.2)	328/75	19.4 (15.8, 23.7)	313/83	21.6 (17.8, 26.1)
10	232/170	43.8 (39.0, 48.9)	251/141	36.5 (31.9, 41.5)	226/166	43.2 (39.0, 48.3)
15	160/235	60.6 (55.7, 65.4)	176/217	56.2 (51.3, 61.2)	169/219	57.0 (52.1, 62.0)
20	133/258	66.5 (61.8, 71.1)	139/250	64.8 (60.0, 69.5)	127/262	68.2 (63.5, 72.8)
25	112/279	71.9 (67.4, 76.3)	106/282	73.1 (68.6, 77.4)	100/290	75.5 (71.1, 79.7)
30	87/303	78.1 (73.9, 82.1)	90/298	77.2 (72.9, 81.3)	78/306	79.7 (75.5, 83.6)
45	53/335	86.3 (82.7, 89.5)	54/334	86.8 (83.2, 89.9)	47/337	87.8 (84.3, 90.8)
60	35/354	91.2 (88.2, 93.8)	31/355	92.0 (89.0, 94.4)	37/348	90.9 (87.7, 93.5)
75	25/363	93.6 (90.8, 95.7)	24/362	93.8 (91.1, 95.9)	31/353	91.9 (88.9, 94.4)
90	16/372	95.9 (93.6, 97.5)	17/369	95.6 (93.2, 97.3)	23/362	94.3 (91.6, 96.3)
105	10/378	97.4 (95.5, 98.7)	12/374	96.9 (94.8, 98.3)	16/368	95.8 (93.5, 97.5)
120	8/380	97.9 (96.1, 99.0)	7/379	98.2 (96.5, 99.2)	11/374	97.4 (95.4, 98.7)
150	5/383	98.7 (97.2, 99.5)	4/382	99.0 (97.5, 99.7)	5/379	98.7 (97.1, 99.5)

Supplementary Table 3. Maternal Serious Adverse Events

	GAP in upright position N = 388			Upright position N = 386			Routine practice N = 384			p-value	Adjusted Risk Ratio[c] (95% confidence interval)		
	N	n	%	N	n	%	N	n	%		GAP vs Routine practice	Upright versus routine practice	GAP vs upright
Maternal Serious Adverse Events (episodes)	388	24	6.2	386	3	0.8	384	6	1.6	<0.0001	2.88 (1.16, 7.15)	0.50 (0.13, 1.88)	5.80 (1.96, 17.16)
Minor[a]	388	8	2.1	386	0	0.0	384	3	0.8	-	-	-	-
Postpartum haemorrhage, retained products of conception	388	4	1.0	386	2	0.5	384	2	0.5	-	-	-	-
Anaemia	388	7	1.8	386	0	0.0	384	1	0.3	-	-	-	-
Sepsis	388	2	0.5	386	0	0.0	384	0	0.0	-	-	-	-
CS complications – wound infection, spinal headache	388	3	0.8	386	1	0.3	384	0	0.0	-	-	-	-

[a] Minor conditions includes: urinary tract infection, second degree perineal laceration, unspecified tachycardia, pelvic and perineal pain, urinary retention
